# A case report of plaque psoriasis comorbid with hidradenitis suppurativa, hepatitis B, and colorectal cancer treated with xeligekimab

**DOI:** 10.3389/fimmu.2025.1651568

**Published:** 2025-10-10

**Authors:** Jun Ma, Binbin Hu, Jiayao Pan, Lunfei Liu

**Affiliations:** Department of Dermatology, The Fourth Affiliated Hospital of School of Medicine, and International School of Medicine, International Institutes of Medicine, Zhejiang University, Yiwu, China

**Keywords:** psoriasis, comorbidities, hidradenitis suppurativa, colorectal cancer, hepatitis B, biologics

## Abstract

**Background:**

Psoriasis and hidradenitis suppurativa are chronic inflammatory skin diseases with common pathogenesis, such as the involvement of IL-17A, which also plays an essential role in the development and metastasis of colorectal cancer. Xeligekimab, a novel IL-17A inhibitor, offers a targeted therapeutic approach for these conditions.

**Case presentation:**

A 43-year-old male presented with a 12-year history of plaque psoriasis and hidradenitis suppurativa. Previous treatment with topical corticosteroids, Calcipotriol Betamethasone Ointment and acitretin provided poor control of psoriasis, resulting in significant quality-of-life impairment. His comorbidities include chronic hepatitis B managed with 10-year antiviral therapy and metastatic colorectal cancer treated with synchronous resection of liver metastases during primary tumor surgery 6 years ago (no recurrence).

**Results:**

After 28 weeks of xeligekimab treatment, the patient experienced significant improvement in symptoms and skin lesions. The evaluation indicators demonstrated a sustained decline: PASI decreased from 51 to 0.8, BSA from 87% to 6%, DLQI from 20 to 1, and Hurley staging improved to Grade I. During follow-up, imaging and tumor marker testing revealed no signs of tumor and hepatitis B progression.

**Conclusion:**

Xeligekimab demonstrated significant efficacy and acceptable safety over 28 weeks in this complex case of psoriasis with concurrent hidradenitis suppurativa, chronic hepatitis B, and metastatic colorectal cancer. Extended follow-up is ongoing to evaluate long-term outcomes, while larger prospective studies are warranted to validate biologic therapy for such multimorbid presentations.

## 1 Introduction

Psoriasis is a chronic inflammatory systemic skin disease characterized by erythematous scaly plaque formation and is often comorbid with other diseases such as cardiovascular disease, other inflammatory skin diseases, and tumors. Recently, research on the pathogenesis of psoriasis has revealed a strong connection between the disease and IL-17. The pathogenic mechanism of IL-17A is initiated by the aberrant activation of Th17 cells. In the skin, local microenvironmental stimuli induce dendritic cells to secrete factors such as IL-23, which promote the differentiation of CD4^+^T cells into Th17 cells and result in the substantial release of IL-17A. Upon binding to receptors on keratinocytes, IL-17A activates the NF-κB and MAPK signaling pathways, thereby driving excessive proliferation of keratinocytes and the secretion of pro-inflammatory cytokines (e.g., IL-6, IL-8) and chemokines (e.g., CCL20). This process recruits neutrophils and T cells to the lesion site, establishing a synergistic vicious cycle of “inflammatory cytokine secretion - immune cell infiltration - abnormal epidermal hyperplasia” in conjunction with other cytokines such as IL-22 and TNF-α. Ultimately, this cascade leads to characteristic pathological changes, including acanthosis (1, 2). Among them, IL-17A and IL-23 are the key factors in the pathogenesis of psoriasis. A clinical study has demonstrated that psoriasis patients treated with secukinumab (an IL-17A antibody) for 12 weeks exhibited a reversal of plaque histopathology, along with a significant reduction in the levels of upstream cytokines IL-23 and IL-17A (3). Furthermore, in patients with nail psoriasis, treatment with IL-17A inhibitors (secukinumab and ixekizumab) over 24 weeks resulted in significantly lower PASI, BSA, DLQI, and NAPSI scores compared to baseline, accompanied by marked improvement in skin lesions (4). Biologic therapies targeting these cytokines have gradually become an indispensable part of the treatment of psoriasis (5).

Hidradenitis suppurativa is also a chronic inflammatory skin disease characterized by recurrent episodes of painful nodules, abscesses, sinus tracts, and scar formation (2). Studies in recent years have confirmed that its pathogenesis shares commonalities with psoriasis, such as both including the involvement of TNF-α and IL-17A (6). The study conducted by Alexa et al. revealed that bimekizumab, a dual inhibitor of IL-17F and IL-17A originally developed for psoriasis treatment, exhibited significant therapeutic efficacy in patients with moderate-to-severe hidradenitis suppurativa. Notably, the therapeutic benefits were sustained for up to 48 weeks (7). In addition, it has been shown that elevated serum levels of IL-17A may be associated with the development and metastasis of many tumors, especially colorectal cancer (8, 9). IL-17A has the potential to be a new target for inhibiting tumor metastasis and recurrence (10).

While biologics are traditionally contraindicated in patients with malignancies and hepatitis B, emerging evidence supports the safety of IL-17A inhibitors in these populations (11, 12). This case demonstrates xeligekimab—a novel fully humanized anti-IL-17A IgG4 monoclonal antibody (China-approved, August 2024)—effectively managing concurrent psoriasis, hidradenitis suppurativa, hepatitis B, and metastatic colorectal cancer, providing clinical validation for this paradigm shift.

## 2 Case report

### 2.1 Patient information

The patient, male, 43 years old, has had recurrent erythematous plaques with scales all over the body, along with multiple nodular abscesses and sinus tracts on the trunk and scalp for 12 years. He also has chronic hepatitis B managed with 10-year antiviral therapy and underwent synchronous resection of liver metastases during colorectal cancer surgery 6 years ago, with no recurrence since. Previous treatment of psoriasis with topical corticosteroids, Calcipotriol Betamethasone Ointment provided poor control. Then the patient was switched to system treatment with acitretin 40mg/day for more than one year result in failed response (PASI improvement <50%). Concomitant therapies included entecavir 0.5 mg/day (for HBV).Despite psoriasis symptoms significantly compromising quality of life, biologic agents had not been attempted due to concerns regarding the patient’s history of malignancy and chronic hepatitis B.

### 2.2 Clinical findings and diagnostic assessment

#### 2.2.1 Plaque psoriasis

**Figure 1:**
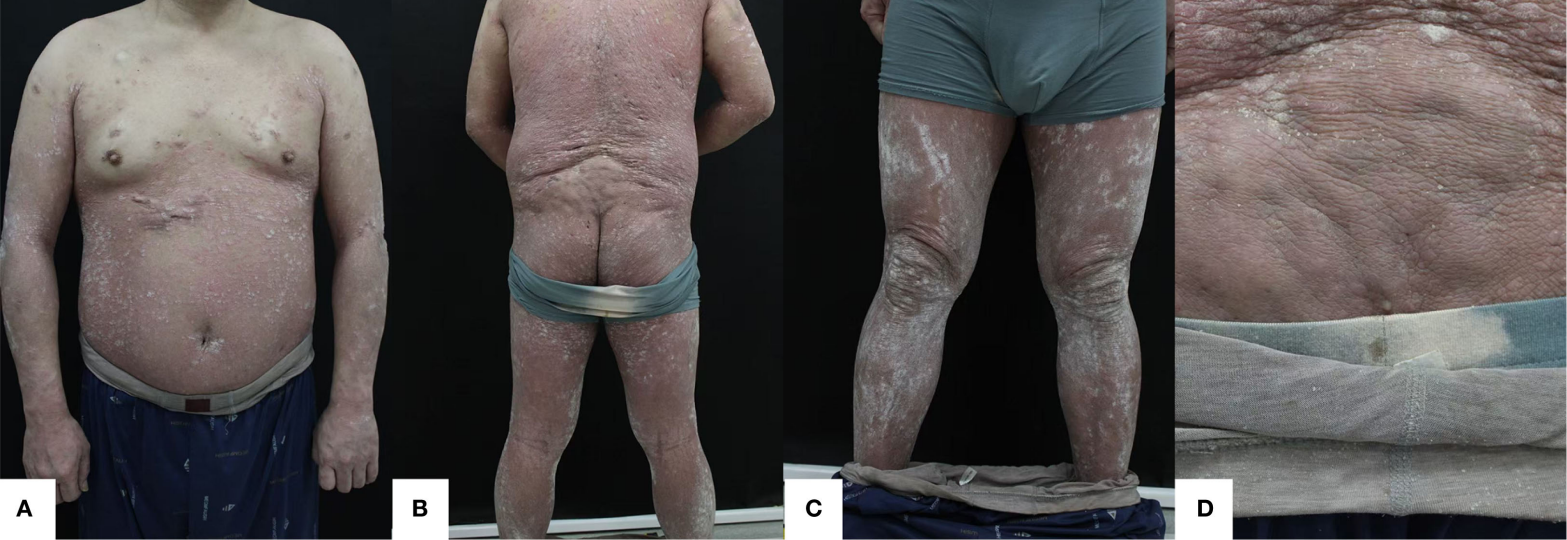
**(A)** Anterior **(B)** Posterior **(C)** Bilateral lower extremities **(D)** Gluteal region. Lesions when not receiving treatment with xeligekimab, PASI:51, BSA:87%, DLQI:20, Hurley: grade II.

Prior to the initiation of treatment, scale-covered erythematous plaque is across the body, with PASI 51, BSA 87%, and DLQI 20 (**Figure 1**).

#### 2.2.2 Hidradenitis suppurativa

Prior to the initiation of treatment, multiple nodules and sinus tracts are on the trunk and scalp, with Hurley classification II (**Figure 1**). On November 20, 2024, imaging diagnosis: Multiple nodules were observed in the subcutaneous soft tissue of both chest walls, along with multiple mildly enlarged lymph nodes in the bilateral axillae.

#### 2.2.3 Post-resection for colorectal cancer with liver metastasis

Synchronous resection of liver metastases was performed during the colorectal cancer surgery. Regular abdominal ultrasounds have been conducted since surgery. There is no recurrence after 6 years of regular follow-up. The most recent abdominal ultrasound (August 20, 2024) revealed: Post partial hepatectomy status; Fatty infiltration in the residual liver parenchyma. The patient’s alpha-fetoprotein (AFP) levels measured 5.66 ng/mL on October 1, 2024 (8 weeks prior to initiation) and 6.7 ng/mL on November 20, 2024 (Baseline), both falling within our institution’s normal reference range (< 7 ng/mL).

#### 2.2.4 Hepatitis B

He has received long-term antiviral therapy for 10 years. On November 21, 2024, the level of hepatitis B virus (HBV) DNA was measured at < 1.0 × 10^1 IU/mL with a normal reference range of < 20 IU/mL at our institution.

### 2.3 Therapeutic intervention

We finally selected xeligekimab, a novel fully humanized IgG4 monoclonal antibody of IL-17A approved for marketing in China in August 2024.

The decision to use xeligekimab was based on:

(1) Established IL-17A pathway involvement in psoriasis and hidradenitis suppurativa pathogenesis (5, 6); (2) Emerging evidence of IL-17A’s potential role in colorectal cancer modulation (8–10); (3) Its IgG4 structure minimizing immunogenicity risks (23).

Specific treatment plan: The initial administration of xeligekimab occurred on November 25, 2024, 200 mg subcutaneous injections of xeligekimab every two weeks, and then every four weeks after 12 weeks of therapy. Concomitant medications: entecavir 0.5 mg once daily and doxycycline 100 mg twice daily.

### 2.4 Follow-up and outcomes

**Figure 2:**
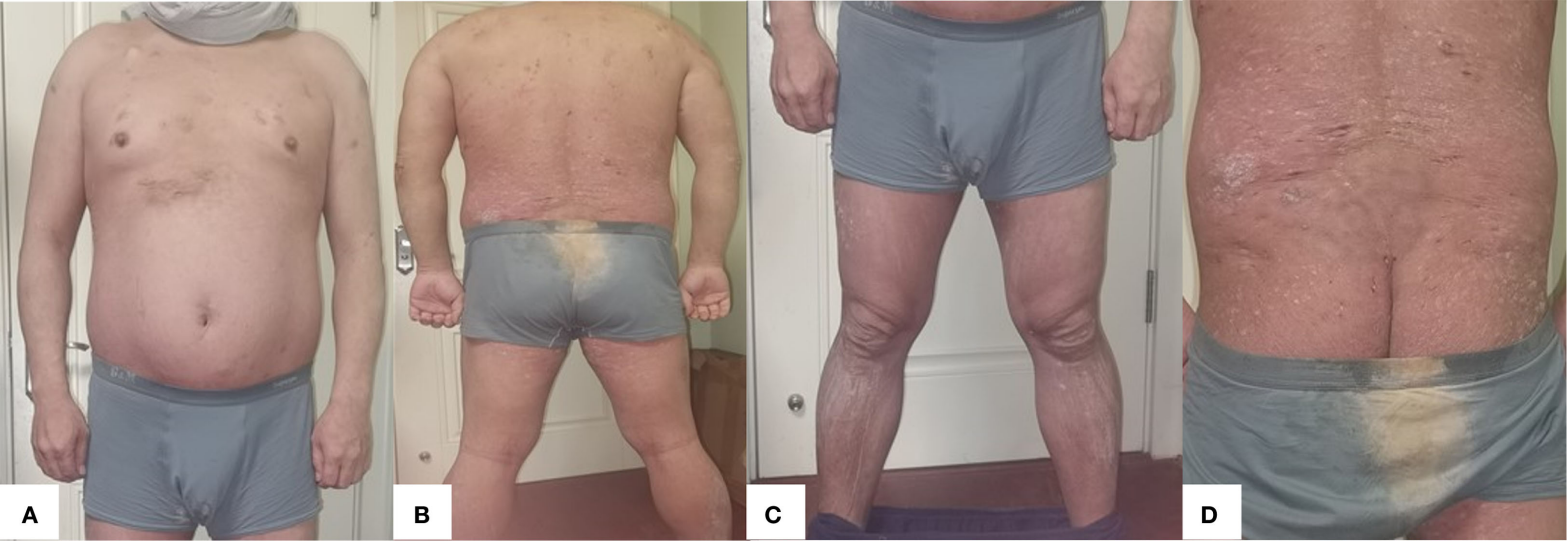
**(A)** Anterior **(B)** Posterior **(C)** Bilateral Lower extremities **(D)** Gluteal region. After 8 weeks of treatment with xeligekimab, PASI: 5.8, BSA: 27%, DLQI: 7, Hurley: Grade I.

**Figure 3:**
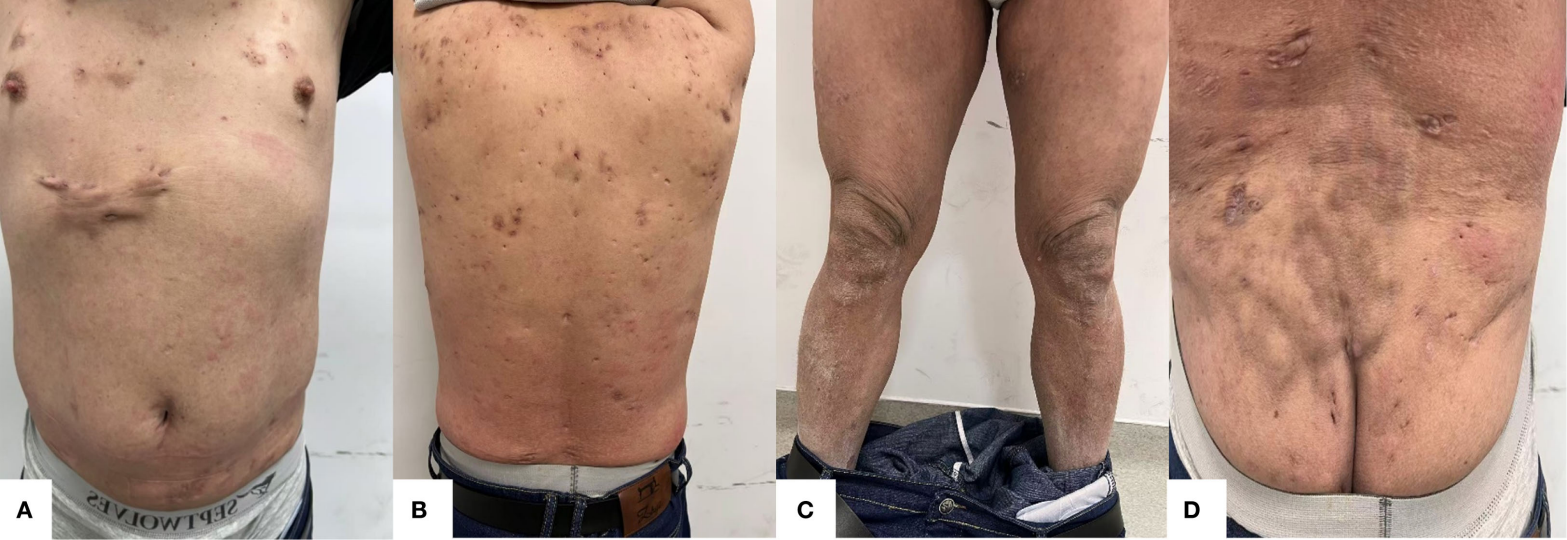
**(A)** Anterior **(B)** Posterior **(C)** Bilateral lower extremities **(D)** Gluteal region. After 28 weeks of treatment with xeligekimab, PASI: 0.8, BSA: 6%, DLQI: 1, Hurley: Grade I.

**Figure 4:**
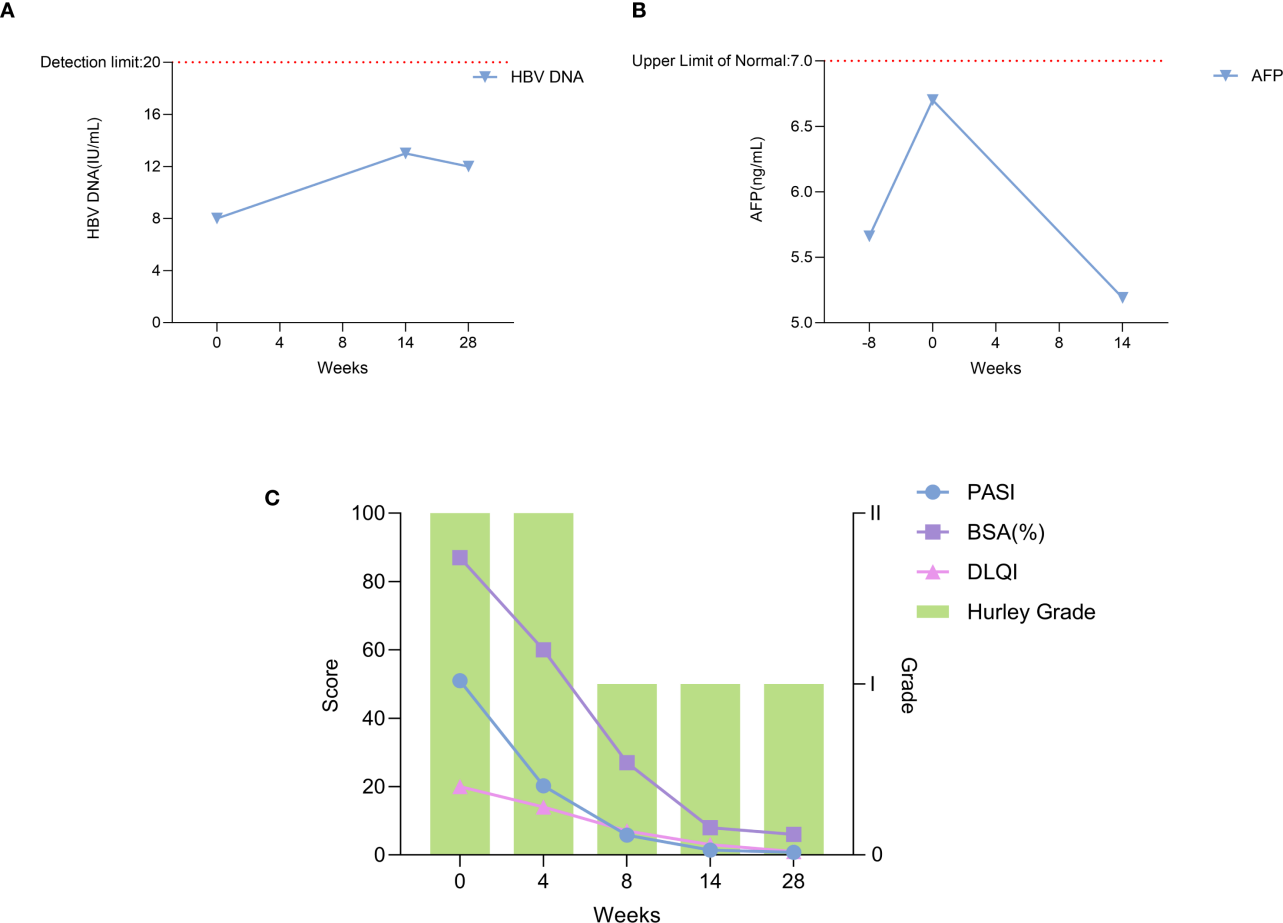
Trends of clinical and laboratory parameters during xeligekimab treatment. **(A)** HBV DNA levels reflecting hepatitis B viral load. **(B)** AFP levels indicating tumor marker alpha-fetoprotein. **(C)** Trends in PASI, BSA, DLQI, and Hurley Stage.

During the 28 weeks of treatment, the patient’s lesions improved significantly, and the scaly erythema virtually disappeared. There are no new rashes, abscesses, nodules, or sinus tracts compared to before, and only one area above the hips still has secretory overflow (**Figures 2**, **3**). We used the Psoriasis Area and Severity Index (PASI), Body Surface Area (BSA), and Dermatology Life Quality Index (DLQI) to assess the patient’s the severity of psoriasis and its impact on the quality of life, and the Hurley classification to assess the severity of the patient’s hidradenitis suppurativa. Throughout the 28-week treatment, all of these indicators dropped (**Figure 4C**; **Table 1**).

**Table 1:** Clinical outcome measures during xeligekimab treatment.

Time (weeks)	PASI	BSA(%)	DLQI	Hurley grade	HBV DNA(IU/mL)	AFP(ng/mL)
-8	–	–	–	–	–	5.66
0	51.0	87	20	II	<1.0 × 10^^1^	6.7
4	20.2	60	14	II	–	–
8	5.8	27	7	I	–	–
14	1.4	8	3	I	<2.0 × 10^^1^	5.19
28	0.8	6	1	I	<2.0 × 10^^1^	–

PASI, Psoriasis Area and Severity Index; BSA, Body Surface Area; DLQI, Dermatology Life Quality Index; AFP, Alpha-Fetoprotein; HBV DNA, Hepatitis B Virus DNA.

On March 5, 2025 (Week 14 of treatment), carcinoembryonic antigen (CEA) was measured at 6.18 ng/mL, AFP was 5.19 ng/mL, and HBV DNA was < 2.0 × 10^1 IU/mL. Abdominal ultrasound revealed: Post partial hepatectomy status; Fatty infiltration in the remnant liver; Calcification in the right hepatic lobe. No new lesions were detected compared with the previous ultrasound (August 20, 2024). At the 28th week of treatment, HBV DNA testing confirmed levels < 2.0 × 10^1 IU/mL. During follow-up, imaging and tumor marker testing revealed no signs of tumor and hepatitis B progression (**Figures 4A, B**).There were no adverse reactions such as hyperuricemia, hyperlipidemia, or injection site reactions.

## 3 Discussion

This complex case—involving plaque psoriasis, hidradenitis suppurativa, chronic hepatitis B, and metastatic colorectal cancer—presented unique therapeutic challenges. Xeligekimab achieved concurrent inflammatory disease control while maintaining oncological stability, suggesting targeted IL-17A blockade may be feasible in such multimorbid scenarios.

Despite concerns that biologics (e.g., TNF-α inhibitors) may trigger tumor recurrence or HBV reactivation, rendering malignancies and hepatitis B relative contraindications, emerging evidence supports IL-17A inhibitors’ safety in these populations (12–14).Secukinumab significantly improved quality of life without increasing cancer recurrence risk in psoriasis patients with malignancy history (13), while IL-17A blockade showed safety in chronic hepatitis B (12).Thus, when clinically warranted after traditional treatment failure, anti-IL-17A therapy represents a feasible option requiring rigorous benefit-risk assessment and informed consent (11, 15).

There are limited previous case report data on the use of biologics for the treatment of psoriasis comorbid with hidradenitis suppurativa, chronic hepatitis B, and metastatic colorectal cancer. Yen, C.F. et al. successfully treated three cases of psoriasis comorbid with hidradenitis suppurativa using adalimumab (16). In 2018, Lasagnas et al. reported a successful treatment with secukinumab in a 59-year-old woman with plaque psoriasis, hepatitis B, and a 12-year history of breast cancer (17). Additionally, some studies have evaluated the use of ixekizumab in treating linear psoriasis and pediatric generalized pustular psoriasis (18, 19), as well as the successful use of spesolimab in a case of generalized pustular psoriasis during pregnancy (20). Our case extends these findings, demonstrating xeligekimab’s efficacy in this challenging clinical scenario. Notably, in 2024, Yousefian et al. described a case of paradoxical psoriasis emerging in a 25-year-old woman with hidradenitis suppurativa following secukinumab therapy, likely due to compensatory IL-23 overexpression (21). Therefore, to prevent exacerbation or recurrence of psoriasis and its comorbidities, a personalized treatment plan must be carefully tailored for each patient.

Biologic therapy was considered due to suboptimal response to conventional treatments. TNF-α inhibitors were excluded over safety concerns in hepatitis B and malignancy (14, 22). Although head-to-head trials are lacking, xeligekimab achieved PASI75 and PASI100 comparable to secukinumab, with potentially superior PASI100 rates (23–25). As a fully human antibody, xeligekimab may offer safety advantages over ixekizumab (23). Given concomitant hidradenitis suppurativa, IL-17 inhibitors appear more effective than IL-23 agents (26). TYK2/PDE4 inhibitors demonstrated significantly inferior efficacy versus IL-17 blockers, with unproven efficacy in hidradenitis suppurativa (27, 28).

Building on these reports, our case further highlights the potential of biologics in managing complex cases of psoriasis with significant comorbidities. The dramatic improvement in our patient’s condition—with PASI reduction from 51 to 0.8, BSA from 87% to 6%, and DLQI from 20 to 1—demonstrates xeligekimab’s potent efficacy. Importantly, the treatment addressed all three conditions simultaneously without adverse effects on hepatitis B viral load or tumor markers, supporting its safety in this complex clinical context.

This case report also has certain limitations, including a relatively short follow-up period and the inherent nature of single-case studies. Further investigations, such as randomized controlled trials, are warranted to confirm the efficacy and safety of xeligekimab in treating psoriasis with complex comorbidities.

## 4 Conclusion

This patient demonstrated significant efficacy and acceptable safety with xeligekimab over 28 weeks of treatment. We will conduct extended longitudinal follow-up to evaluate long-term safety and efficacy outcomes. The benefit-risk profile of biologics in such complex cases requires further validation through larger prospective studies.

## Data Availability

The original contributions presented in the study are included in the article/Supplementary Material. Further inquiries can be directed to the corresponding author.

## References

[1] Blauvelt A Chiricozzi A. The immunologic role of IL-17 in psoriasis and psoriatic arthritis pathogenesis. Clin Rev Allergy Immunol. (2018) 55:379–90. doi: 10.1007/s12016-018-8702-3, PMID: 30109481 PMC6244934

[2] Brembilla NC Senra L Boehncke W. The IL-17 family of cytokines in psoriasis: IL-17A and beyond. Front Immunol. (2018) 9:1682. doi: 10.3389/fimmu.2018.01682, PMID: 30127781 PMC6088173

[3] Krueger JG Wharton KAJ Schlitt T Suprun M Torene RI Jiang X. IL-17A inhibition by secukinumab induces early clinical, histopathologic, and molecular resolution of psoriasis. J Allergy Clin Immunol. (2019) 144:750–63. doi: 10.1016/j.jaci.2019.04.029, PMID: 31129129

[4] Yan X Shi M Wang B Zeng L Wang H Shi J. Targeting nail psoriasis: IL-17A inhibitors demonstrate site-specific superiority over IL-23 inhibitor in a 24-week dermoscopy-guided real-world cohort. Front Immunol. (2025) 16:1573715. doi: 10.3389/fimmu.2025.1573715, PMID: 40264783 PMC12011727

[5] Griffiths CEM Armstrong AW Gudjonsson JE Barker JNWN. Psoriasis. Lancet. (2021) 397:1301–15. doi: 10.1016/S0140-6736(20)32549-6, PMID: 33812489

[6] Saunte DML Jemec GBE. Hidradenitis suppurativa: advances in diagnosis and treatment. Jama. (2017) 318:2019–32. doi: 10.1001/jama.2017.16691, PMID: 29183082

[7] Kimball AB Jemec GBE Sayed CJ Kirby JS Prens E Ingram JR. Efficacy and safety of bimekizumab in patients with moderate-to-severe hidradenitis suppurativa (BE HEARD I and BE HEARD II): two 48-week, randomised, double-blind, placebo-controlled, multicentre phase 3 trials. Lancet. (2024) 403:2504–19. doi: 10.1016/S0140-6736(24)00101-6, PMID: 38795716

[8] Lereclus E Tout M Girault A Baroukh N Caulet M Borg C. A possible association of baseline serum IL-17A concentrations with progression-free survival of metastatic colorectal cancer patients treated with a bevacizumab-based regimen. BMC Cancer. (2017) 17:220. doi: 10.1186/s12885-017-3210-z, PMID: 28347290 PMC5368920

[9] Blake SJ Teng MWL. Role of IL-17 and IL-22 in autoimmunity and cancer. Actas Dermosifiliogr. (2014) 105 Suppl 1:41–50. doi: 10.1016/S0001-7310(14)70017-1, PMID: 25398491

[10] Song M Liang J Wang L Li W Jiang S Xu S. IL-17A functions and the therapeutic use of IL-17A and IL-17RA targeted antibodies for cancer treatment. Int Immunopharmacol. (2023) 123:110757. doi: 10.1016/j.intimp.2023.110757, PMID: 37579542

[11] Mastorino L Dapavo P Avallone G Merli M Cariti C Rubatto M. Biologic treatment for psoriasis in cancer patients: should they still be considered forbidden? J Dermatol Treat. (2022) 33:2495–502. doi: 10.1080/09546634.2021.1970706, PMID: 34409918

[12] Thatiparthi A Martin A Liu J Egeberg A Wu JJ. Biologic treatment algorithms for moderate-to-severe psoriasis with comorbid conditions and special populations: A review. Am J Clin Dermatol. (2021) 22:425–42. doi: 10.1007/s40257-021-00603-w, PMID: 33861409 PMC8051287

[13] Pellegrini C Esposito M Rossi E Gisondi P Piaserico S Dapavo P. Secukinumab in patients with psoriasis and a personal history of Malignancy: A multicenter real-life observational study. Dermatol Ther (Heidelb). (2022) 12:2613–26. doi: 10.1007/s13555-022-00797-9, PMID: 36169883 PMC9588094

[14] Kridin K Zirpel H Mruwat N Ludwig RJ Thaci D. Evaluating the risk of infections under interleukin 23 and interleukin 17 inhibitors relative to tumour necrosis factor inhibitors - A population-based study. J Eur Acad Dermatol Venereol. (2023) 37:2319–26. doi: 10.1111/jdv.19328, PMID: 37466275

[15] Patel S Patel T Kerdel FA. The risk of Malignancy or progression of existing Malignancy in patients with psoriasis treated with biologics: case report and review of the literature. Int J Dermatol. (2016) 55:487–93. doi: 10.1111/ijd.13129, PMID: 26711080

[16] Yen C Huang Y Chi C. Concomitant psoriasis and hidradenitis suppurativa responsive to adalimumab therapy: A case series. Indian J Dermatol Venereol Leprol. (2021) 87:223–26. doi: 10.4103/ijdvl.IJDVL_455_18, PMID: 31389375

[17] Lasagni C Bigi L Conti A Pellacani G. Successful therapy of plaque-type psoriasis with secukinumab in patients with multiple comorbidities treated with previous biologic therapies. J Dermatol Treat. (2018) 29:5–08. doi: 10.1080/09546634.2018.1543843, PMID: 30403898

[18] Christov S Ohm F Augustin M Wagner JN. Successful treatment of linear psoriasis with the IL-17a-antagonist ixekizumab: A case report. Psoriasis (Auckl). (2025) 15:23–8. doi: 10.2147/PTT.S499039, PMID: 39866910 PMC11762445

[19] Esposito M Antonetti P Vagnozzi E De Berardinis A Bertelli R Brancati F. Ixekizumab as a successful treatment in pediatric generalized pustular psoriasis. Ital J Pediatr. (2025) 51:41. doi: 10.1186/s13052-024-01835-2, PMID: 39934839 PMC11816982

[20] Zhang S Peng G Zhang Y Luo L Li Y Luo J. Successful treatment of generalized pustular psoriasis during pregnancy with secukinumab in a patient hypersensitive to spesolimab: a case report. J Dermatol Treat. (2025) 36:2474495. doi: 10.1080/09546634.2025.2474495, PMID: 40112342

[21] Yousefian F Griffith V Stallings A Rupley K. A case of secukinumab-induced psoriasis in a 25-year-old man with improved hidradenitis suppurativa. Jaad Case Rep. (2024) 53:90–2. doi: 10.1016/j.jdcr.2024.08.027, PMID: 39823051 PMC11736156

[22] Kuo MH Tseng C Tseng K Lu M Tung C Chen N. The relationship between TNF-alpha inhibitor potency and HBV reactivation in patients with rheumatic disorders. Liver Int. (2025) 45:e70152. doi: 10.1111/liv.70152, PMID: 40418092

[23] Cai L Jiang C Zhang G Fang H Wang J Li Y. A multicentre randomized double-blind placebo-controlled phase III study of the efficacy and safety of xeligekimab (GR1501) in patients with moderate-to-severe plaque psoriasis. Br J Dermatol. (2024) 191:336–43. doi: 10.1093/bjd/ljae062, PMID: 38366639

[24] Pan J Chang X Wang L Miao G Jin Q Guo N. Use of biologics in patients with psoriasis - A retrospective analysis based on real-world data. Skin Res Technol. (2024) 30:e13550. doi: 10.1111/srt.13550, PMID: 38174801 PMC10765354

[25] Cai L Zhang J Yao X Gu J Liu Q Zheng M. Secukinumab demonstrates high efficacy and a favorable safety profile over 52 weeks in Chinese patients with moderate to severe plaque psoriasis. Chin Med J (Engl). (2020) 133:2665–73. doi: 10.1097/CM9.0000000000001163, PMID: 33060370 PMC7647502

[26] Heidari N Heidari A Eghbali S Pishraft-Sabet H Hajikarim-Hamedani A Ghane Y. The role of interleukin inhibitors in the treatment of hidradenitis suppurativa; a systematic review of clinical trials. Autoimmun Rev. (2025) 24:103818. doi: 10.1016/j.autrev.2025.103818, PMID: 40268126

[27] Ighani A Partridge ACR Shear NH Lynde C Gulliver WP Sibbald C. Comparison of management guidelines for moderate-to-severe plaque psoriasis: A review of phototherapy, systemic therapies, and biologic agents. J Cutan Med Surg. (2019) 23:204–21. doi: 10.1177/1203475418814234, PMID: 30463416

[28] Huang C Huang I Tai C Chi C. Biologics and small molecule inhibitors for treating hidradenitis suppurativa: A systematic review and meta-analysis. Biomedicines. (2022) 10:1303. doi: 10.3390/biomedicines10061303, PMID: 35740325 PMC9220298

